# Molecular Screening for Nigericin Treatment in Pancreatic Cancer by High-Throughput RNA Sequencing

**DOI:** 10.3389/fonc.2020.01282

**Published:** 2020-07-31

**Authors:** Zhihua Xu, Guanzhuang Gao, Fei Liu, Ye Han, Chen Dai, Sentai Wang, Guobang Wei, Yuting Kuang, Daiwei Wan, Qiaoming Zhi, Ye Xu

**Affiliations:** ^1^Department of General Surgery, The First Affiliated Hospital of Soochow University, Suzhou, China; ^2^Department of Gastroenterology, The First Affiliated Hospital of Soochow University, Suzhou, China; ^3^Department of Colorectal Surgery, Fudan University Shanghai Cancer Center, Shanghai Medical College, Shanghai, China

**Keywords:** nigericin, high-throughput sequencing, long non-coding RNA, bioinformatics, pancreatic cancer

## Abstract

**Objectives:** Nigericin, an antibiotic derived from *Streptomyces hygroscopicus*, has been proved to exhibit promising anti-cancer effects on a variety of cancers. Our previous study investigated the potential anti-cancer properties in pancreatic cancer (PC), and demonstrated that nigericin could inhibit the cell viabilities in concentration- and time-dependent manners via differentially expressed circular RNAs (circRNAs). However, the knowledge of nigericin associated with long non-coding RNA (lncRNA) and mRNA in pancreatic cancer (PC) has not been studied. This study is to elucidate the underlying mechanism from the perspective of lncRNA and mRNA.

**Methods:** The continuously varying molecules (lncRNAs and mRNAs) were comprehensively screened by high-throughput RNA sequencing.

**Results:** Our data showed that 76 lncRNAs and 172 mRNAs were common differentially expressed in the nigericin anti-cancer process. Subsequently, the bioinformatics analyses, including Gene ontology (GO) and Kyoto Encyclopedia of Genes and Genomes (KEGG) analysis, coding and non-coding co-expression network, cis- and trans-regulation predictions and protein-protein interaction (PPI) network, were applied to annotate the potential regulatory mechanisms among these coding and non-coding RNAs during the nigericin anti-cancer process.

**Conclusions:** These findings provided new insight into the molecular mechanism of nigericin toward cancer cells, and suggested a possible clinical application in PC.

## Introduction

Ductal adenocarcinoma of the exocrine pancreas, commonly known as pancreatic cancer (PC), is a highly aggressive malignancy with few effective therapies. At the time of diagnosis, −20% of PC patients are considered eligible for surgery and of these, about a half undergoes successful resection (1). But unfortunately, a majority of patients with PC are diagnosed at advanced stages, at which patients can hardly receive surgical R0 resection (2) with a 5-years survival rate of 3% and a median survival of <6 months (3–5). In spite of significant advances in surgical care, chemotherapy and radiotherapy, no effective systemic therapy for the aggressive pathology of this cancer is available. One of the reasons for the treatment failures is due to resistance to chemotherapy or radiotherapy (6). Thus, novel therapeutic agents are needed to treat PC and improve the overall survival of patients with this disease.

Up to date, standard treatments for cancer involve chemotherapy with anti-tumor antibiotic. Adriamycin, an orally administered DNA alkylating agent, has been the most potent chemotherapy applied in clinic, in addition to surgical excision. Bleomycin had been emerged as another vital chemotherapeutic agent in many types of cancer, including Hodgkin lymphoma (7), testicular cancer (8), and squamous-cell carcinoma (9). In the previous study, we firstly identified salinomycin as a novel identified cancer stem cells (CSCs) killer in gastric cancer cells (10). Afterwards, we also found that salinomycin could specifically target on cisplatin-resistant colorectal cancer cells by accumulating reactive oxygen species (11). Recently, Moxifloxacin and ciprofloxacin induced cell apoptosis and S-phase arrest via ERK activation in PC (12). Similar anti-cancer influences of antibiotic on PC cells were found also in recent literatures. In 2012, Yadav et al. demonstrated that gatifloxacin possessed anti-proliferative activity against PC cell lines by causing S/G2 phase cell cycle arrest without induction of apoptosis through p21, p27, and p53 dependent pathway (13). They also investigated the effect of moxifloxacin and ciprofloxacin on survival and proliferation of PC cell lines, and found that both were able to suppress the proliferation of PC cells and induce apoptosis through the similar mechanism (12).

Nigericin is a monocarboxylic polyether antibiotic potassium ionophore that is widely used as a coccidiostatic agent in chickens (14). In 1972, the effects of nigericin on intracellular pH, glycolysis, and K^+^ concentration of ehrlich ascites tumor cells were firstly reported (15). Since then, emerging evidence confirmed the promising anti-cancer activity of nigericin in a variety of cancers, including prostate cancer (16), nasopharyngeal carcinoma (17), bladder cancer (18), chronic lymphocytic leukemia cells (19), and lung cancer (20). In 2004, Vaupel et al. reported that nigericin could inhibit breast cancer stem cells at least 100 times more effectively than paclitaxel in mice (21). Zhou et al. demonstrated that nigericin could suppress the colorectal cancer metastasis through inhibition of epithelial-mesenchymal transition (EMT) (22). Recently, our study explored the circular RNA (circRNA) expression profiles after nigericin exposure on PC cells through bioinformatics method, and discussed the potential function of nigericin in PC (23). However, our knowledge of nigericin, which correlates with long non-coding RNA (lncRNA) and mRNA in PC, has not been studied yet.

In this study, we attempted to ascertain the specific activities of nigericin on human PC cell lines and investigate its possible molecular mechanism in PC. The continuously varying molecules (lncRNAs and mRNAs) were displayed by the high-throughput sequencing. Through analyzing the aberrant expressions of lncRNAs and mRNAs as well as their potential relationships, the molecular mechanisms of nigericin treatment on PC were discussed.

## Materials and Methods

### Cell Culture and Reagents

Human PC cell lines (PANC-1) were purchased from Shanghai Institute of Biochemistry and Cell Biology at the Chinese Academy of Sciences (Shanghai, China). Cells were cultured in Dulbecco's Modified Eagle Medium (DMEM, Gibco) supplemented with 10% fetal bovine serum (FBS, Gibco) at 37°C in a humidified incubator containing 5% CO_2_. Cells were in the logarithmic phase of growth for all experiments. Nigericin was purchased from Sigma Aldrich (USA). The stock solutions (100 mmol/L) were prepared with dimethyl sulfoxide (DMSO) and stored at −20°C.

### High-Throughput RNA Sequencing Analysis

PANC-1 cells were exposed to a proper concentration of nigericin (5 μmol/L) according to the results of 50% inhibitory concentration (IC50) for different time periods (0, 8, 16, or 32 h), and then total RNA was extracted from cells, respectively. The quantity and integrity of total RNAs were measured by the NanoDrop™ ND-2000 (Thermo Fisher Scientifc, Scotts Valley, CA, USA) and Agilent Bioanalyzer 2100 (Agilent Technologies, Santa Clara, CA, USA), respectively. lncRNAs and mRNAs were quantitatively analyzed by Shanghai OE Biotech (Shanghai, China). After removal of ribosomal RNA and then constructing a library, a high-throughput RNA sequencing was performed. The clean reads were aligned to the reference genome by Bowtie2 (http://bowtie-bio.sourceforge.net/bowtie2/manual.shtml). For unmapped reads, the junctions were picked out using back-splice algorithm. Finally, lncRNAs and mRNAs were verified with software developed by Shanghai OE Biotech, which were considered as the reference sequence for further analysis.

### Differentially Expressed lncRNA and mRNA Screen and Clustering Analysis

Differentially expressed lncRNAs and mRNAs were detected by the negative binomial distribution test based on the DESeq package. These lncRNAs and mRNAs with statistical significance were screened with *p* < 0.05, false discovery rate (FDR) <0.05 and fold change (FC) more than 2.0. Difference integration analysis (Venn analysis) was used to show the often characteristic elements among these 3 compared groups (0 vs. 8 h, 0 vs. 16 h, 0 vs. 32 h). The common differentially expressed lncRNAs and mRNAs were showed in pies with different colors. The non-supervised hierarchical clustering of the differentially expressed lncRNAs and mRNAs was used in the form of heat map to display the expression patterns of the differential lncRNAs and mRNAs between different groups.

### Quantitative Real-Time Polymerase Chain Reaction (qRT-PCR) Validation

Total RNA from cell lines was extracted using Trizol solution (Invitrogen, USA) and converted into cDNA by using M-MLV reverse transcriptase (Invitrogen, USA). The quantities and qualities of isolated RNAs were evaluated using absorbance measurements at 260 and 280 nm. Then reverse transcription (RT) was performed in a 20 μl reaction system using the ReverAid First Stand cDNA Synthesis (Thermo Scientific, Mountain View, CA, USA). RT-PCR with SybGreen I (Generay Bio Co., Shanghai, China) was performed using the 7500 real-time PCR system (Applied Biosystems, Hayward, CA, USA) with the follow program: initial denature at 95°C for 10 min, followed by 40 cycles of 95°C for 10 s and 60°C for 60 s. β-actin was used as control. Results were harvested in three independent wells. The sequences of primers were listed as follows: LINC00667:6 (F: 5′CCCGACTTTTTGATGCAGGC3′; R: 5′CCCGACTGTTTCCTACCCAC3′), Lnc-HMGN1-1:12 (F: 5′GATCATGGCTCTCTCTGCCA3′; R: 5′AGCTGTTACATACGGCCCAC3′), Lnc-LRRC24-2:1 (F: 5′GATTCGCTGGACGATCGCA3′; R: 5′CCTGTAAAGGGAACGCGTCA3′), Lnc-AC007952.1.1-3:1 (F: 5′GCGAGAAAGGTTTTCGCCTC3′; R: 5′ACAATAGGAGGTGCCACACA3′), Lnc-CCNB1IP1-1:2 (F: 5′TGTCCCTTGGGAAGGTCTGA3′; R: 5′CCCGTTCTCTGGGAACTCAC3′), GADD45A (F: 5′GAGAGCAGAAGACCGAAAGGA3′; R: 5′CACAACACCACGTTATCGGG3′), HBP1 (F: 5′TCATCACCATTGGAAGGAGGA3′; R: 5′TTGCACCATCCCAAATCATCA3′), SESN2 (F: 5′AAGGACTACCTGCGGTTCG3′; R: 5′CGCCCAGAGGACATCAGTG3′), KIF20A (F: 5′TTGAGGGTTAGGCCCTTGTTA3′; R: 5′GTCCTTGGGTGCTTGTAGAAC3′), TOP2A (F: 5′ACCATTGCAGCCTGTAAATGA3′; R: 5′GGGCGGAGCAAAATATGTTCC3′), and β-actin (F: 5′CCTGTACGCCAACACAGTGC3′; R: 5′ATACTCCTGCTTGCTGATCC3′).

### Gene Ontology (GO) and Kyoto Encyclopedia of Genes and Genomes (KEGG) Pathway Analysis

GO analysis was conducted to construct meaningful annotations of genes and gene products in a wide variety of organisms through DAVID database (http://david.abcc.ncifcrf.gov). Our GO analysis provided the ontology of defined terms which represented gene product properties, and covered three domains: cellular components, biological process and molecular function. The top 10 enriched GO terms, which were derived from the common differentially expressed mRNAs and ranked by enrichment score, were presented. KEGG pathway analysis was also adopted to map differentially expressed mRNAs in different biological pathways. The top 20 enriched pathways among the four groups ranked by enrichment score were calculated and shown.

### lncRNA-mRNA Co-expression Network

To elucidate the potential functions of differentially expressed lncRNAs and explore the relationships between common differentially expressed lncRNAs and mRNAs, the lncRNA-mRNA co-expression network was constructed. For each differentially expressed lncRNA, we calculated the Pearson Correlation of its expression value with the expression value of each differentially expressed mRNA. It was considered to be correlated when the *P-*value of the correlation coefficient of lncRNAs and mRNAs' expression value was not higher than 0.05, and the absolute value of correlation was not <0.7. A total of 66 lncRNAs and mRNAs were selected to generate the network map.

### Cis- and Trans-Regulation Predictions

As previous studies defined, a cis-regulator is the one that exerts its functions on the neighboring genes which were located at the same chromosome. lncRNAs are showed that they can regulate gene expressions in a cis-manner (24, 25). The cis-regulation regions in this study were identified by the following procedures. For each common differentially expressed lncRNA, we identified the mRNAs as “cis-regulated mRNAs” when: (1) the mRNAs loci were within 100 k windows up- and downstream of the given lncRNA. (2) the Pearson Correlation of lncRNA-mRNA expression was statistically significant (*p*-value of correlation ≤0.05).

For trans-regulation prediction, we focused on the manner that lncRNAs played their functions via transcription factors (TFs). The TF-lncRNA and TF-lncRNA-gene network were constructed, respectively. For each differentially expressed lncRNAs, the coding genes co-expressed with them were calculated, and the significance of the gene enrichment in each TF entry was calculated using the hypergeometric distribution test method. The result of the calculation returned a *p*-value that was enriched for significance. A small *p*-value indicated that gene has been enriched in the TF entry. We calculated the intersection of lncRNAs co-expressed gene sets with target gene sets of transcription factor/chromatin regulated complex, and calculated the degree of enrichment of the intersection through hypergeometric distribution method. Then we obtained the TFs which were significantly associated with lncRNAs, and identified possible transcription factor/chromatin regulated factors that might play a combined regulatory role with lncRNAs. Subsequently, we used the analysis results of hypergeometric distribution to visualize the network diagram. Through the hypegeometric distribution calculation, each lncRNA got multiple TF-lncRNA relationship pairs, and each TF-lncRNA pair was the results of enrichments of multiple genes. According to the *p*-value from small to large sort, the top 200 lines of regulatory relationships were used to construct the TF-lncRNA binary relationship network, and the top 10 lines of regulatory relationships were applied to construct the TF-lncRNA-gene ternary relationship network.

### Protein-Protein Interaction (PPI) Network Construction

The Search Tool for the Retrieval of Interacting Genes (STRING, http://string.embl.de/) database was used to construct the PPI network of the common differentially expressed mRNAs. The PPI network was subsequently visualized using Cytoscape. Confidence score ≥0.7 was set as the cut-off criterion, and Molecular Complex Detection (MCODE) was conducted to screen modules of PPI network with degree cutoff = 2, node score cutoff = 0.2, k-core = 2, and max. depth = 100. In addition, a sub-network was constructed by selecting several candidate mRNAs.

### Statistical Analysis

Statistically significant differences among groups were estimated by the Student's *t-*test using SPSS 19.0 software (SPSS Inc.). *P* < 0.05 was considered to be statistically significant.

## Results

### Differentially Expressed lncRNA and mRNA Profile by Sequencing

**Figure 1:**
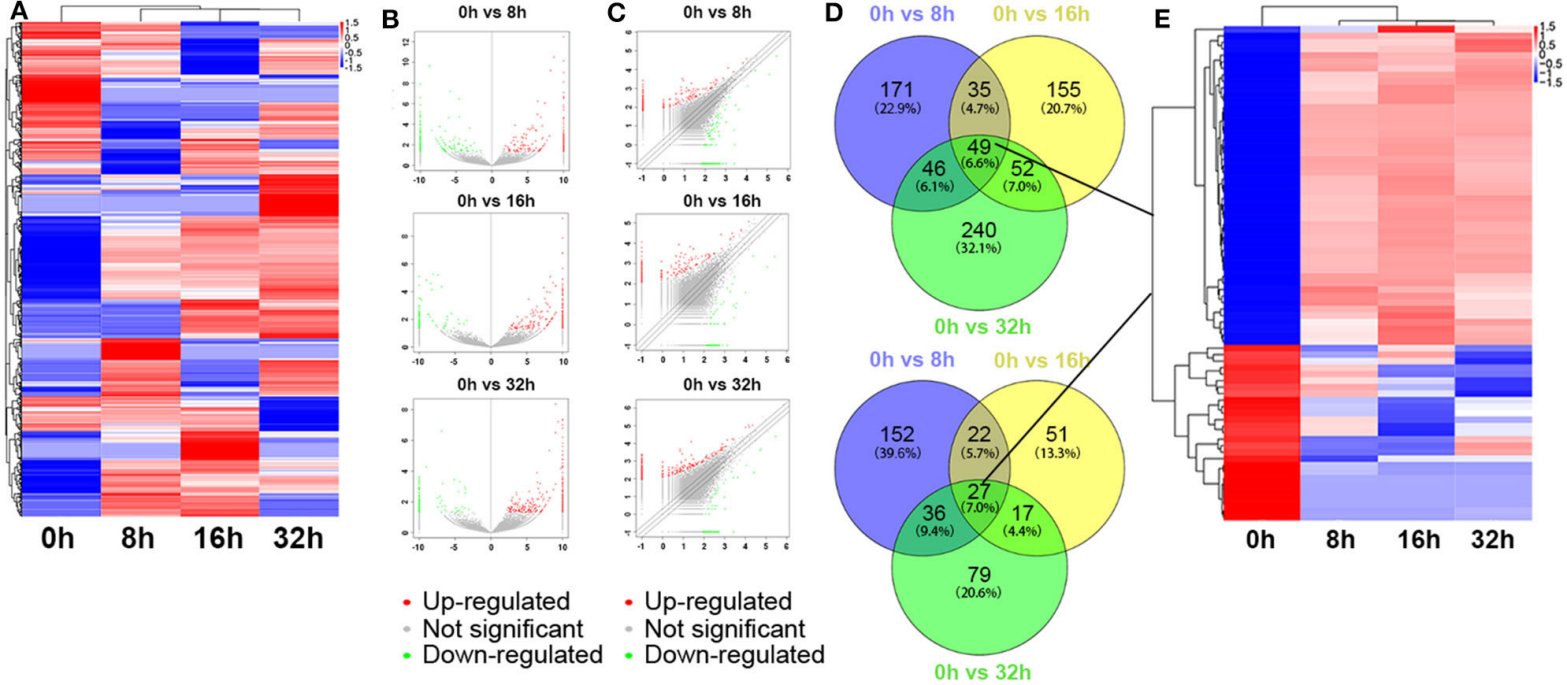
Differentially expressed lncRNA profile by sequencing. **(A)** The cluster heatmap showed all differentially expressed lncRNAs at different time points (0, 8, 16, and 32 h). **(B,C)** The volcano and scatter plots presented differentially expressed lncRNAs between different compared groups (0 h vs. 8 h, 0 h vs. 16 h, 0 h vs. 32 h), respectively. **(D,E)** Venn analysis analyzed the common differentially expressed lncRNAs among the three compared groups, and described them as a cluster heatmap.

**Figure 2:**
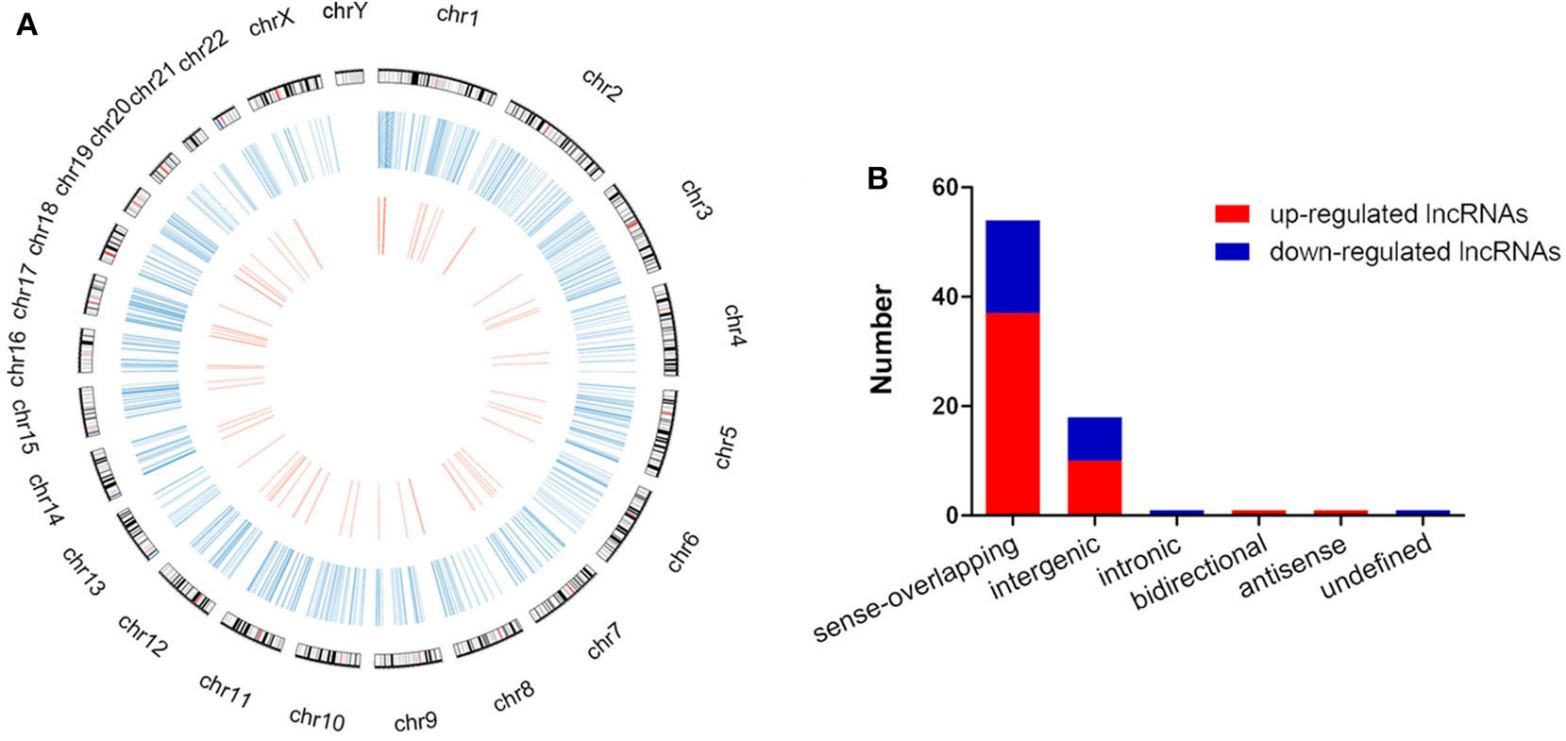
Distribution and classification of the common differentially expressed lncRNAs. **(A)** The circos plot showed the distribution of lncRNAs on human chromosomes. The outermost layer of circos plot was chromosome map of the human genome (the black and white bars were chromosome cytobands, the red bars represented centromeres). The larger inner circle (blue) represented all differentially expressed lncRNAs at different time points, and the smaller inner circle (red) indicated the 76 common differentially expressed lncRNAs with fold change > 2.0, *p* < 0.05 and FDR < 0.05. **(B)** These 76 common differentially expressed lncRNAs were classified into 6 types, including sense-overlapping, intergenic, intronic, bidirectional, antisense and undefined.

The global expression profile of lncRNAs at 4 different time points (0, 8, 16, and 32 h) was determined by a custom sequencing platform. In total, 118,314 lncRNAs were detected, and hundreds of lncRNAs showed differential expressions in each group of different time points (Figure 1A). Three compared groups were set according to the nigericin-treated time points (0 vs. 8 h, 0 vs. 16 h, 0 vs. 32 h). Compared to the 0 h group, 538 days-regulated lncRNAs (more than 2 folds) were found in 8 h, of which 301 lncRNAs were up-regulated and 237 ones were down-regulated. Similarly, 408 lncRNAs were differentially expressed in 16 h group with 291 up-regulated and 117 down-regulated ones, compared to the 0 h group. With the change of treatment time at 32 h, 387 differential lncRNAs were up-regulated, and 159 ones were down-regulated. All differentially expressed lncRNAs with statistical significance were selected with *p* < 0.05, FDR < 0.05, and FC > 2.0 (Figures 1B,C). Venn analysis was used to determine the common differentially expressed lncRNAs among the three compared groups. Our data confirmed that 76 common dys-regulated lncRNAs including 49 up-regulated and 27 down-regulated ones might participate in the process of nigericin damage (Figures 1D,E). To systematically predict the function of lncRNAs, lncRNA subgroup analyses were performed. These lncRNAs were widely distributed on all chromosomes except for sex chromosome X (Figure 2A). Moreover, we adapted specific probes for these lncRNAs to classify several kinds of lncRNAs. Among these dys-regulated lncRNAs, there were 71.1% sense-overlapping, 23.7% intergenic, 1.3% intronic, 1.3% bidirectional, 1.3% antisense, and 1.3% undefined (Figure 2B).

**Figure 3:**
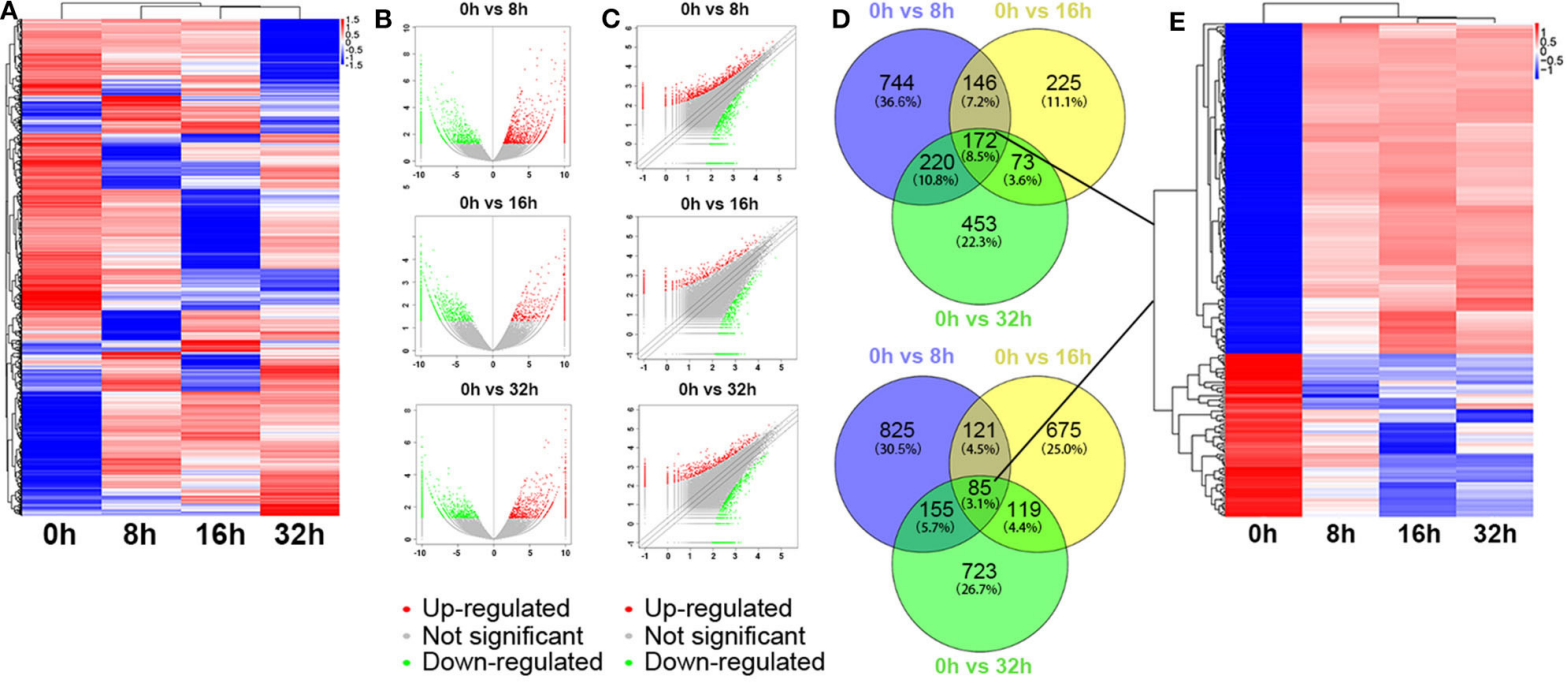
Differentially expressed mRNA profile by sequencing. **(A)** The cluster heatmap showed all differentially expressed mRNAs at different time points (0, 8, 16, and 32 h). **(B,C)** The volcano and scatter plots presented differentially expressed mRNAs between different compared groups (0 vs. 8 h, 0 vs. 16 h, 0 vs. 32 h), respectively. **(D,E)** Venn analysis analyzed the common differentially expressed mRNAs among the three compared groups, and described them as a cluster heatmap.

Besides, the global expression profile of mRNAs was also observed. Among the 98,121 coding transcripts examined, hundreds of coding transcripts were differentially expressed at different time points (Figure 3A). Three compared groups were also set as described above. A total of 2,468 mRNAs exhibited significant differential expression between the 0 and 8 h group, in which 1,282 mRNAs were up-regulated and 1,186 ones were down-regulated. For 0 vs. 16 h group, 616 mRNAs were up-regulated and 1,000 ones were down-regulated. Similarly, for 0 vs. 32 h group, 918 mRNAs were up-regulated and 1,082 ones were down-regulated (Figures 3B,C). All the differentially expressed mRNAs with statistical significance were screened with *p* < 0.05, FDR < 0.05, and FC > 2.0. Venn analysis was also used to determine the common differentially expressed mRNAs among the three compared groups (0 vs. 8 h, 0 vs. 16 h, and 0 vs. 32 h). The results showed that 172 mRNAs were common up-regulated and 85 ones were down-regulated (Figure 3D). As shown in Figure 3E, the common dys-regulated mRNAs were evaluated by the hierarchical clustering analysis.

### Validation of Sequencing Data by qRT-PCR

**Figure 4:**
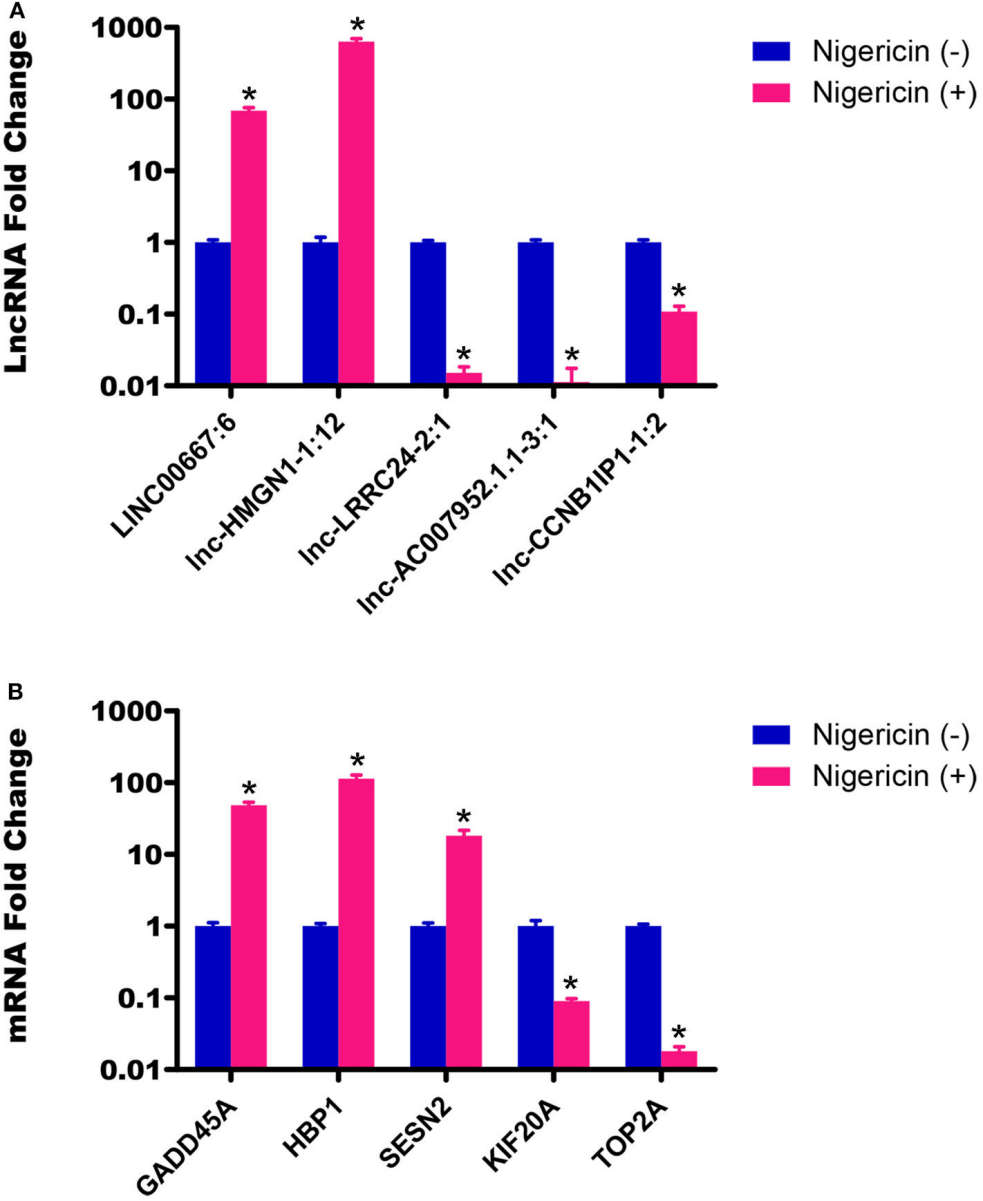
Validation of sequencing data by qRT-PCR. **(A)** The expressions of 5 lncRNAs between the 0 and 32 h group were detected by qRT-PCR. **(B)** The expressions of 5 mRNAs between the 0 and 32 h group were also determined by qRT-PCR (*P < 0.05).

To ensure that our results were reliable, we assessed the expressions of 5 lncRNAs and 5 mRNAs between the 0 and 32 h group by qRT-PCR. Our results showed that the LINC00667:6 and lnc-HMGN1-1:12 were both up-regulated at 32 h, compared to the 0 h group, whereas other 3 lncRNAs (lnc-LRRC24-2:1, lnc-AC007952.1.1-3:1, and lnc-CCNB1IP1-1:2) were down-regulated (Figure 4A, ^*^P < 0.05). Meanwhile, we also chose 5 random cancer-related genes for mRNA detection, and found that GADD45A, HBP1, and SESN2 were significantly up-regulated, whereas KIF20A and TOP2A were down-regulated, compared to the 0 h group (Figure 4B, ^*^P < 0.05). These data were consistent well with the sequencing data, which demonstrated the high reliability and validity of the sequencing expression results.

### GO and KEGG Pathway Analysis

**Figure 5:**
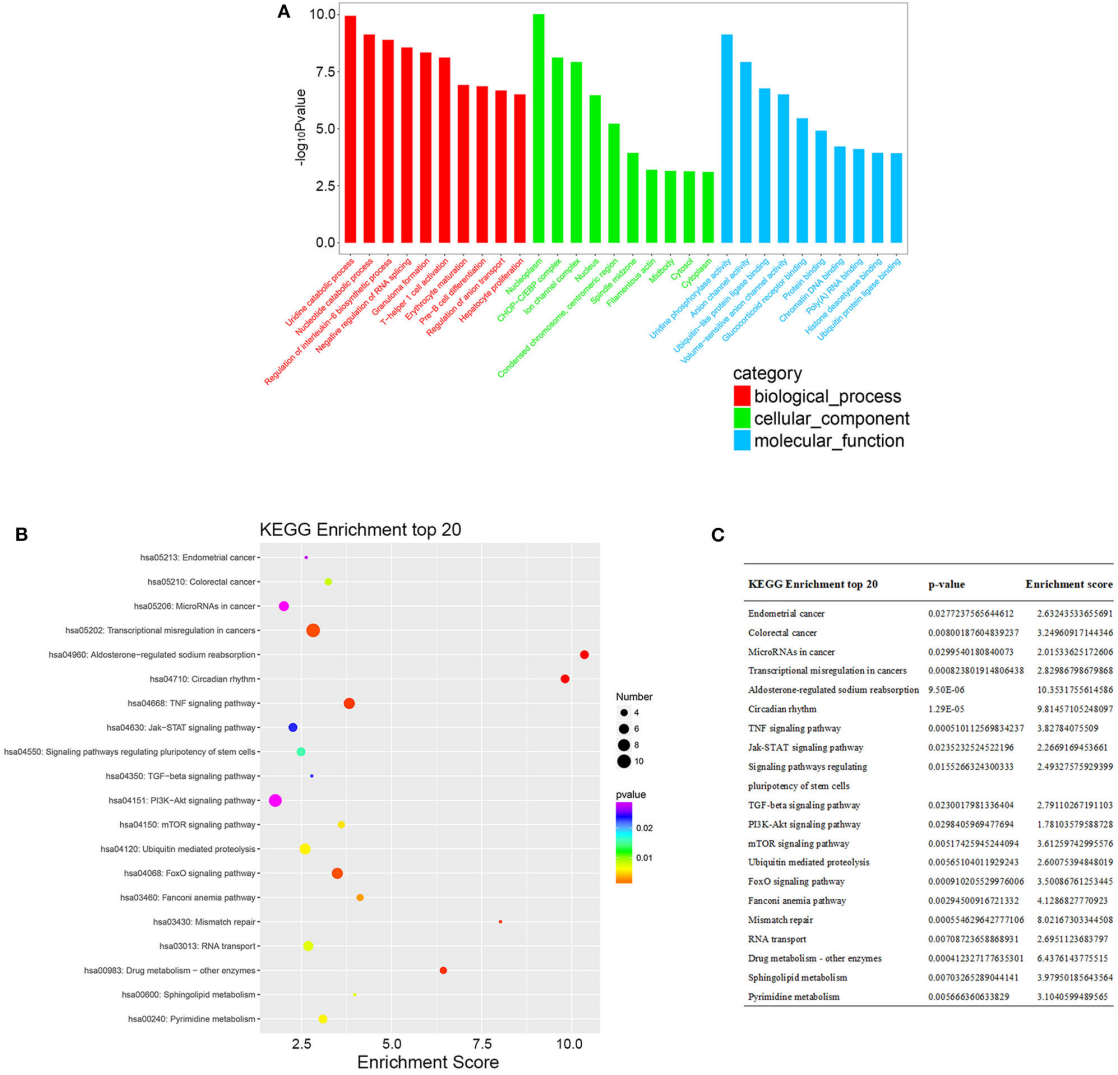
GO and KEGG pathway analysis of the common differentially expressed mRNAs. **(A)** GO analysis was conducted and covered three domains: cellular components, biological process and molecular function. The top 10 enriched GO terms were presented. **(B)** KEGG pathway analysis was also adopted and the top 20 enriched pathways were calculated and shown. **(C)** *P-*value and enrichment score of the top 20 enriched pathways were included and shown.

**Figure 6:**
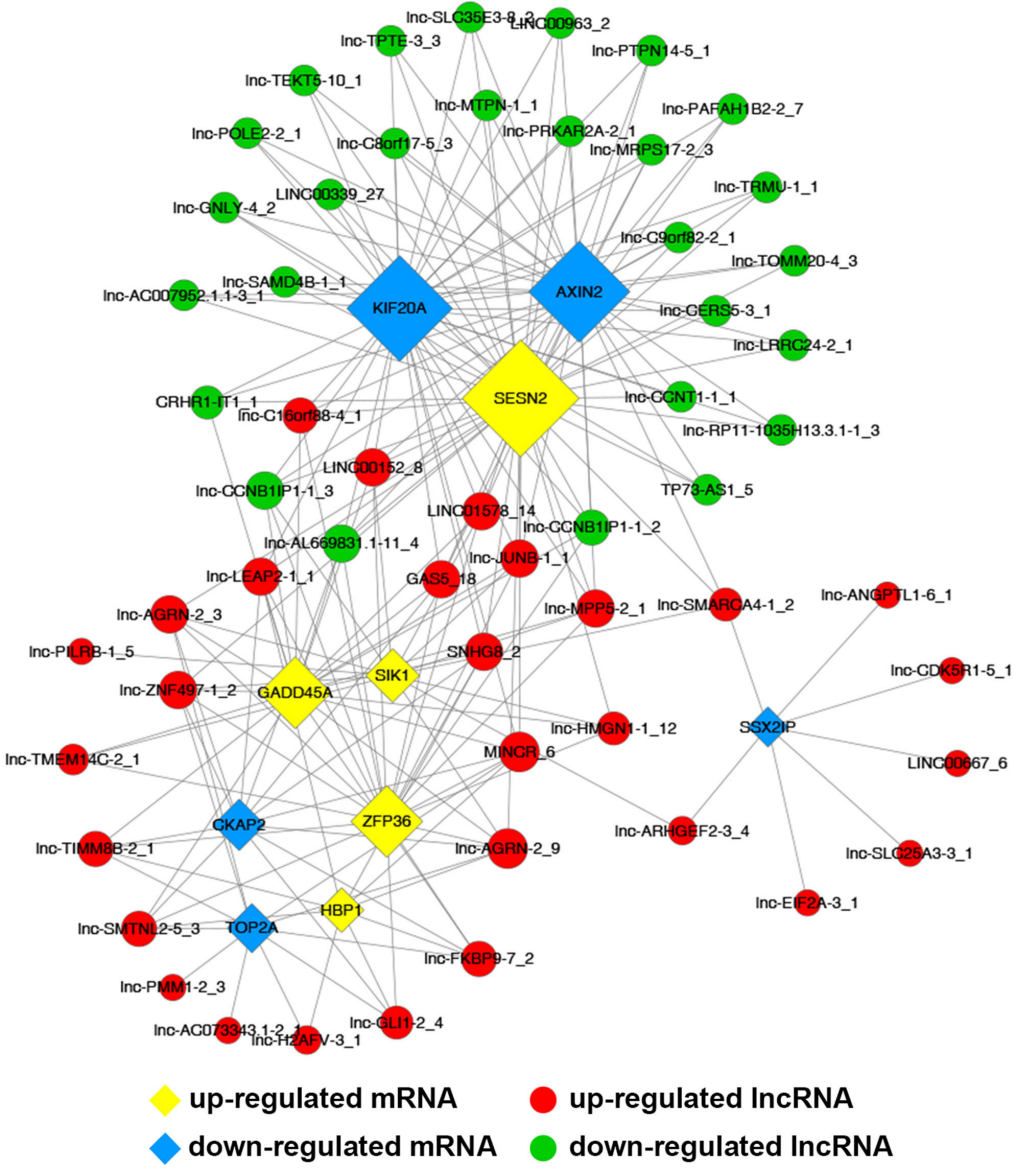
The network between KEGG pathways and their corresponding genes was analyzed, in which 169 pathways and 94 genes were included.

According to the common differentially expressed mRNAs among the three compared groups (0 vs. 8 h, 0 vs. 16 h, 0 vs. 32 h), the GO biological processes classification was calculated. The top 10 GO biological processes such as uridine catabolic process, nucleotide catabolic process and regulation of interleukin-6 biosynthetic process were involved in the nigericin damage. Meanwhile, the top 10 cellular components and molecular functions were also analyzed and presented in Figure 5A. KEGG pathway analysis for the common differentially expressed mRNAs was used to elucidate the pathways related to these mRNAs. Our data showed that differentially expressed mRNAs were significantly enriched in top 20 KEGG signaling pathways, including Aldosterone-regulated sodium reabsorption, Circadian rhythm, Mismatch repair, Drug metabolism-other enzymes, TNF signaling pathway, Transcriptional misregulation in cancers, TGF-beta signaling pathway, PI3K-Akt signaling pathway and so on (Figure 5B). The corresponding *p-*value and enrichment score of the top 20 enrichment pathways were shown in Figure 5C. Using the results of KEGG enrichment analysis of genes, the network between all KEGG pathways and their corresponding genes was analyzed. One hundred sixty-nine pathways and 94 genes were included, and some genes involved in multiple KEGG pathways could be found to provide auxiliary reference for selection of candidate genes. For instance, PRKCA was found to participate in 60 KEGG pathways, including mTOR signaling pathway, PI3K-Akt signaling pathway, MicroRNAs in cancer, Choline metabolism in cancer, Wnt signaling pathway, MAPK signaling pathway, Pancreatic secretion, VEGF signaling pathway, Ras signaling pathway, Pathways in cancer and so on (Figure 6).

### Construction of Coding and Non-coding Co-expression Network

**Figure 7:**
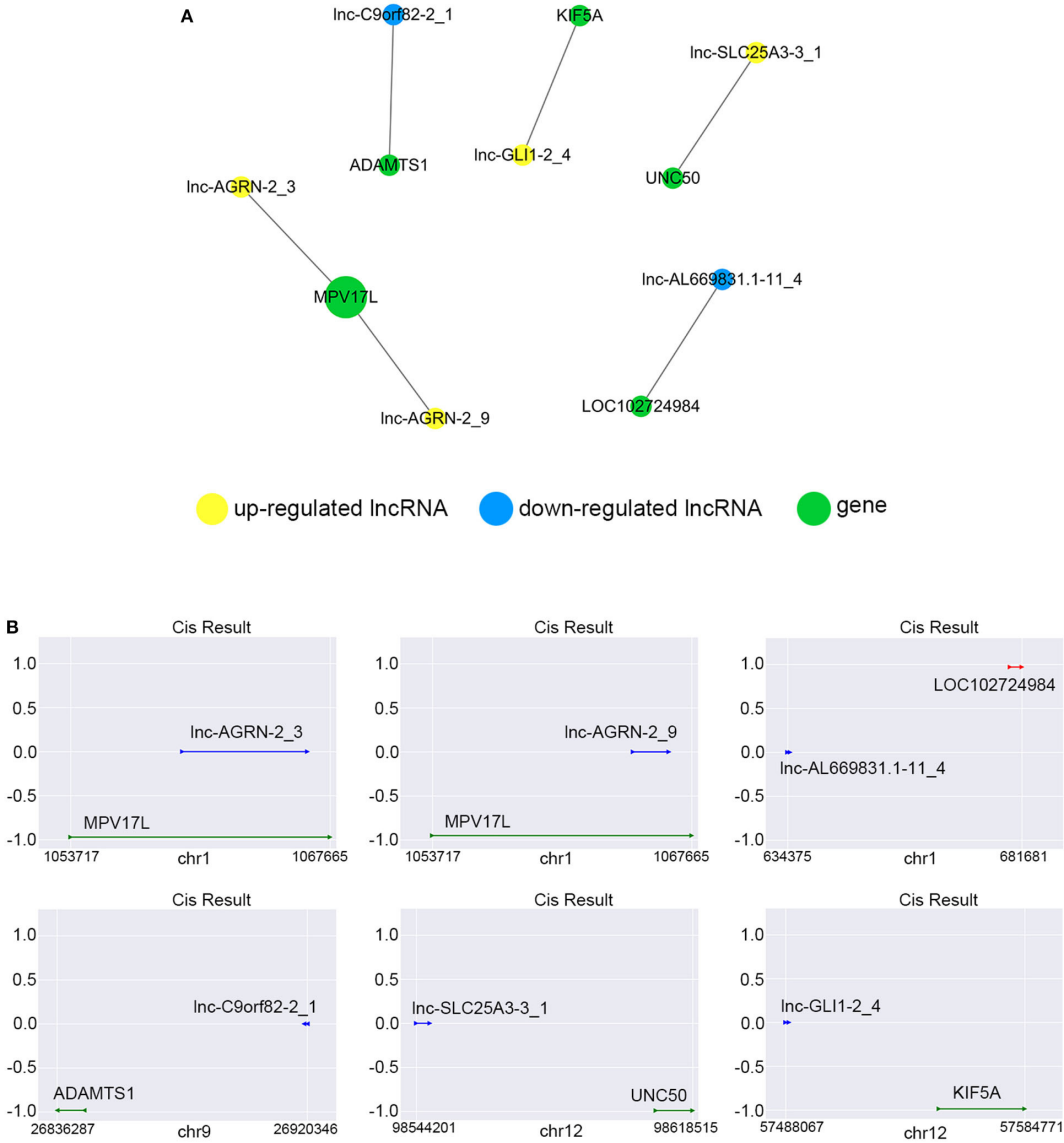
Construction of coding and non-coding co-expression network. **(A)** Common differentially expressed mRNAs (five up-regulated and five down-regulated ones, respectively) were selected to construct the network with their co-expressed common differentially expressed lncRNAs. The network consisted of 66 nodes and 221 connections. **(B)** The cis result of the coding and non-coding co-expression network.

Common differentially expressed mRNAs (five up-regulated and five down-regulated ones, respectively), which were proved to implicate in multiple biological processes including cell cycle, apoptosis, angiogenesis and metastasis, were selected to build this network (Figure 7). The network implied a complex relationship that one gene could correlate with multiple lncRNAs and one lncRNA might also regulate numerous mRNAs in different ways. As shown in Figure 7, up-regulated lnc-AGRN-2_9 was positively correlated with HBP1, GADD45A, SIK1, and SESN2, and negatively associated with TOP2A, CKAP2, while these mRNAs were implicated in apoptosis. Meanwhile, down-regulated SSX2IP, which was involved in tumorigenesis and metastasis, was negatively correlated with lnc-SMARCA4-1_2, lnc-EIF2A-3_1, lnc-SLC25A3-3_1, lnc-ANGPTL1-6_1, lnc-ARHGEF2-3_4, lnc-CDK5R1-5_1, and LINC00667_6. The co-expression network might imply the potential regulatory mechanisms between lncRNAs and mRNAs in the nigericin anti-cancer process.

### Cis-Regulating Function Prediction of lncRNAs

**Figure 8:**
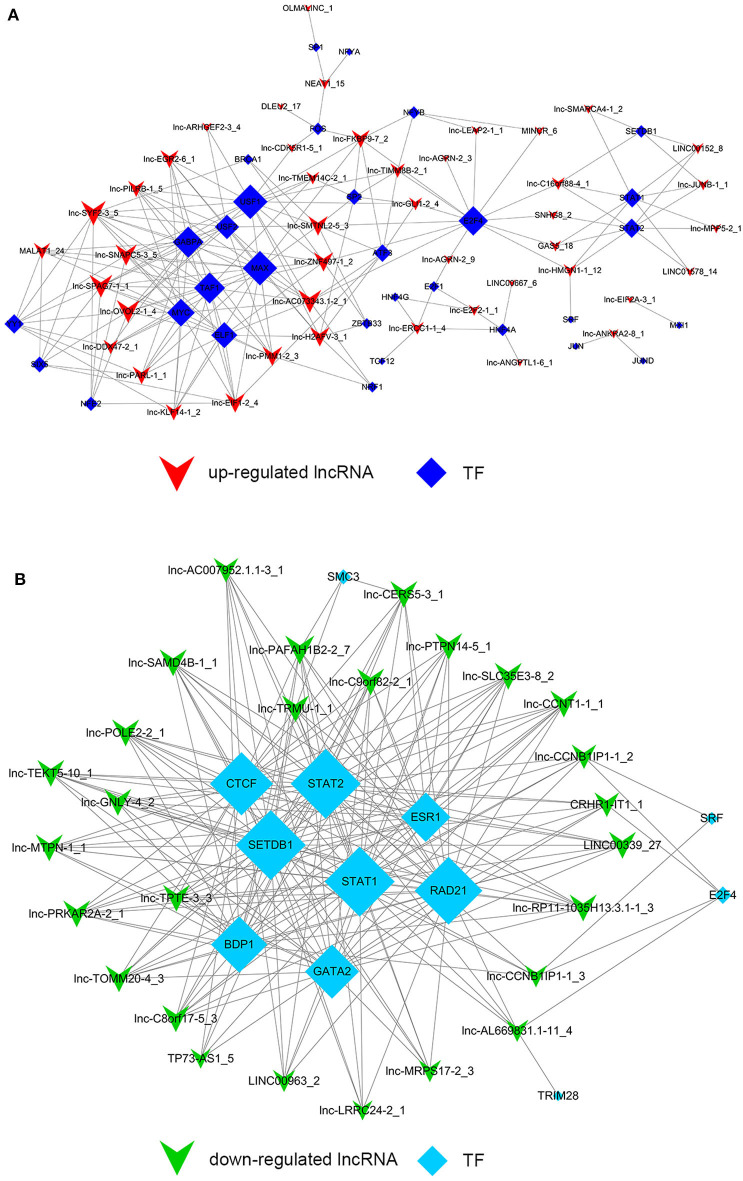
Cis-regulation prediction of lncRNAs. **(A)** Among the 76 common differentially expressed lncRNAs, 6 lncRNAs were found to own neighboring protein-coding genes coding genes. The potential cis-regulation network was described. **(B)** The location and distances between lncRNAs and their nearby coding genes on the chromosome were presented.

We constructed the correlated expression networks to elucidate the relationship between the common differentially expressed lncRNAs and their co-expressed adjacent coding genes. Among all the 76 common differentially expressed lncRNAs, only 6 lncRNAs were found to own neighboring protein-coding genes, and these 6 lncRNAs' potential cis-regulation networks were described in Figure 8A. However, each lncRNA had only one nearby coding gene. For example, lnc-AGRN-2_3 and MPV17L, lnc-AGRN-2_9 and MPV17L, lnc-AL669831.1-11_4 and LOC102724984, lnc-C9orf82-2_1 and ADAMTS1, lnc-GLI1-2_4 and KIF5A, lnc-SLC25A3-3_1 and UNC50 were shown in Figure 8B. The networks might furnish valuable clue for these lncRNAs with nearby coding genes.

### Trans-Regulation of lncRNAs (TF-lncRNA and TF-lncRNA-Gene Network)

**Figure 9:**
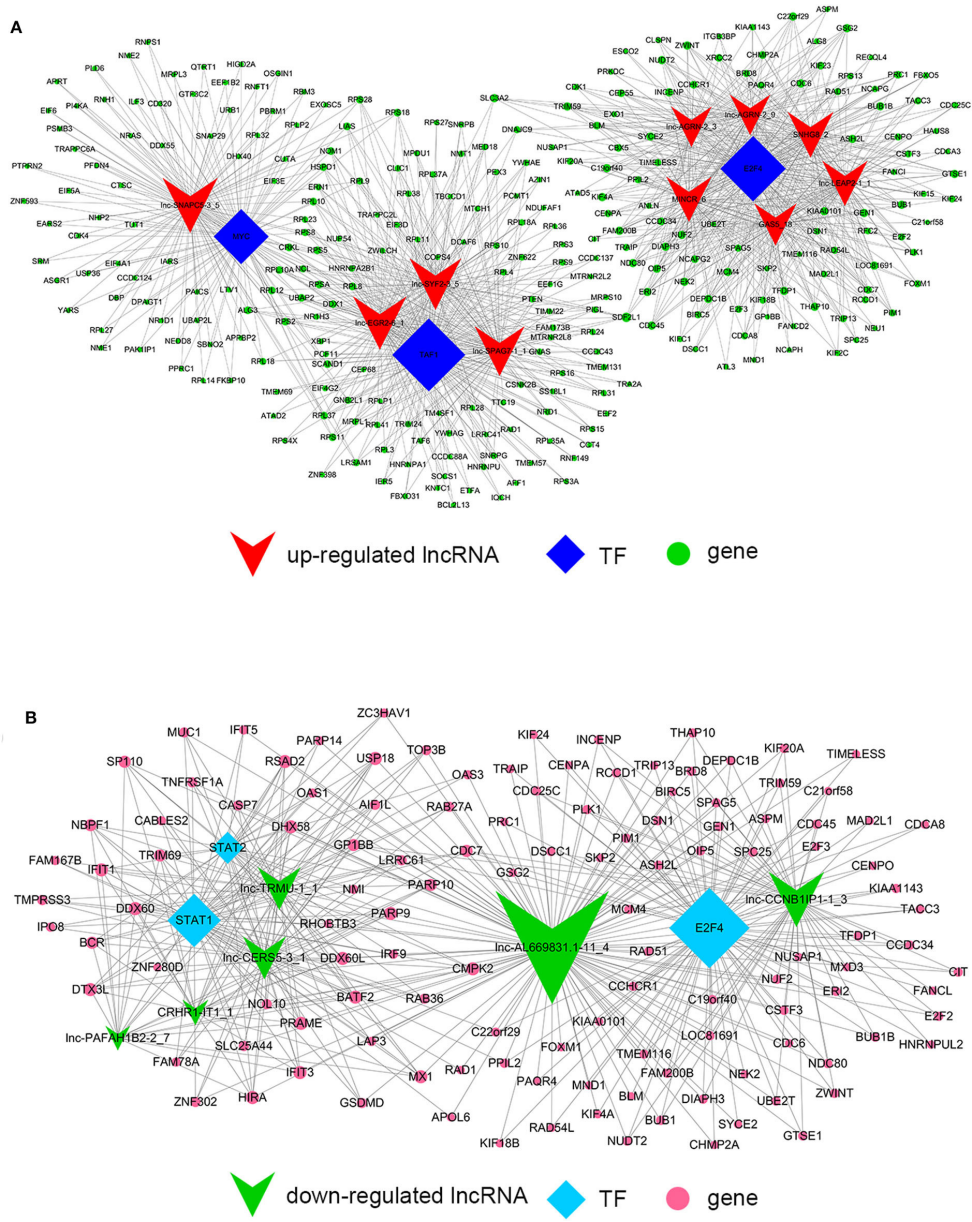
Trans-regulation prediction of lncRNAs (TF-lncRNA binary network). **(A)** Among the 76 common differentially expressed lncRNAs, 44 up-regulated lncRNAs were found to correspond to 31 TFs. **(B)** 27 down-regulated lncRNAs corresponded to 12 TFs.

**Figure 10:**
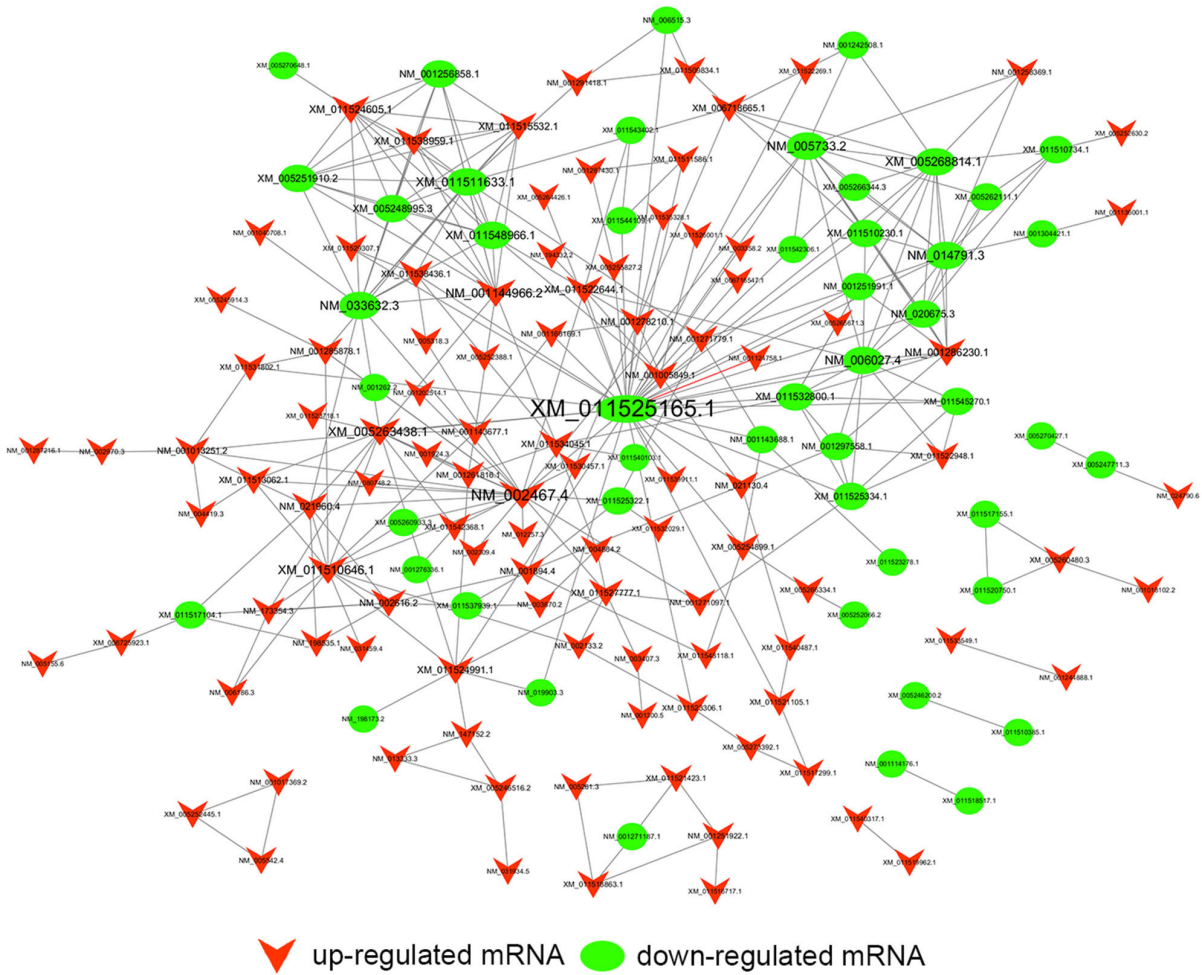
Trans-regulation prediction of lncRNAs (TF-lncRNA-gene ternary network). Target genes were introduced to build TF-lncRNA-gene ternary network. 10 up-regulated lncRNAs correspond to 3 TFs and 283 target genes, while 6 down-regulated lncRNAs were found to associate with 3 TFs and 125 target genes.

Despite the prevalence of lncRNA-mediated cis-regulation, examples of trans-acting lncRNAs have also been reported (25, 26). For trans-regulation prediction, we constructed a co-expression network combined by these common differentially expressed lncRNAs with TFs. With a threshold of *P* < 0.01 and FDR < 0.01, the top 200 closest relationships were selected, while we constructed a TF-lncRNA binary network. The network showed that 44 up-regulated lncRNAs were found to correspond to 31 TFs, and 27 down-regulated lncRNAs corresponded to 12 TFs (Figures 9A,B). Moreover, we introduced target genes to build TF-lncRNA-gene ternary network. 10 up-regulated lncRNAs correspond to 3 TFs and 283 target genes, while six down-regulated lncRNAs were found to associate with 3 TFs and 125 target genes (Figure 10). Interestingly, up to 14 dys-regulated lncRNAs were regulated by 5 TFs, such as MYC, TAF1, E2F4, STAT1, and STAT2. The results implied that these TFs might also participate in the nigericin anti-cancer damage.

### PPI Network Construction

**Figure 11:**
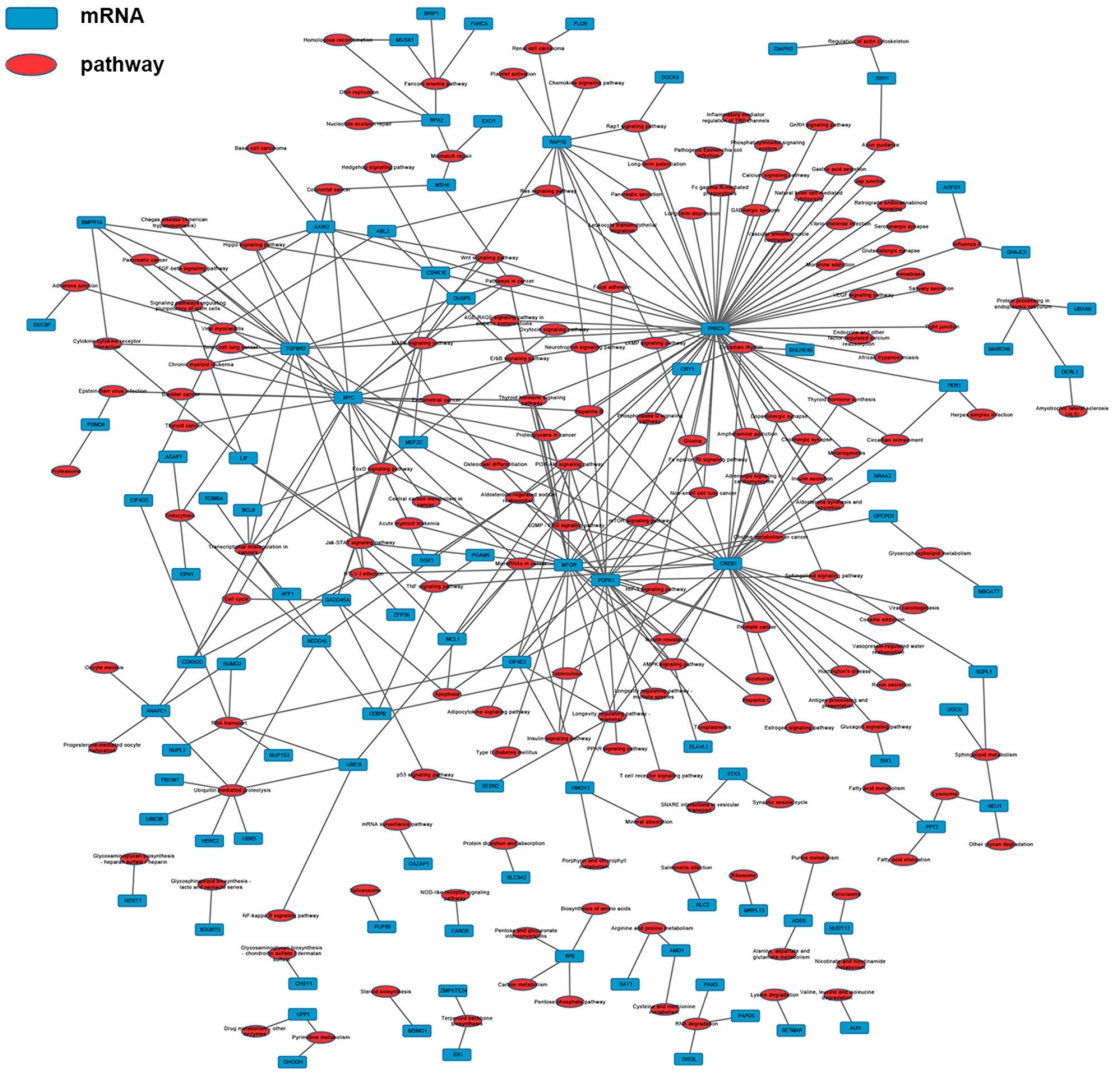
Protein-protein interaction (PPI) network. A total of 152 common differentially expressed genes (103 up-regulated genes and 49 down-regulated ones) were filtered into the network complex. The lines represented the interaction relationship among these genes.

As shown in Figure 11, a total of 152 genes of the 257 common differentially expressed genes were filtered into the PPI network containing 152 nodes and 644 edges. The nodes with high degrees were defined as hub proteins in the PPI networks and degree >10 was set as the cut-off criterion. In this network, a total of 12 nodes were selected as hub proteins, including TOP2A, MYC, ANAPC1, FBXW7, KIF20A, MTOR, CREB1, EXO1, MELK, NEDD4L, RACGAP1, and HERC2. The most significant hub proteins were TOP2A (degree = 40) and MYC (degree = 21). This network exhibited the interactions among these genes which might play a significant role in the nigericin treatment.

## Discussion

Recently, the anti-cancer effect of nigericin has drawn increasing attentions, and its molecular mechanisms toward cancer cells were gradually discovered. A newly study by Yakisich et al. demonstrated that nigericin might be used in a co-therapy model of lung cancer in combination with other chemotherapeutic agents (27). Coincidentally, our lab also implied that Wnt/β-catenin signaling might have an essential role in colorectal cancer progression, and nigericin exerted anti-cancer effects on colorectal cancer cells by directly targeting the β-catenin destruction complex (28). Furthermore, our recent study has proved the potential toxicity of nigericin on human PC, and revealed the molecular mechanism of nigericin toward PC cells from the perspective of circRNA (23). However, the knowledge of nigericin needs to be further elucidated from multiple perspectives.

Along with the deepening of research on PC, numerous lncRNAs have shown to be essential for the tumorigenesis and progression by serving as tumor oncogenes or suppressors. In 2016, Li et al. found that long non-coding RNA metastasis-associated lung adenocarcinoma transcript 1 (MALAT1) could facilitate the advanced progression of PC by promoting autophagy *in vitro* (29). lncRNA myocardial infarction-associated transcript (MIAT) was found remarkably increased in PC tissues and cell lines, and PC patients with high MIAT levels had poor prognosis than those with low MITA levels (30). In contrast, Lnc-PCTST might exhibit as a potential tumor suppressor in PC, which inhibited cell proliferation, invasion, tumorigenesis and EMT by modulating TACC-3 (31). To further explore the anti-cancer mechanism of nigericin, we used high-throughput and bioinformatics methods to predict the changes of coding and non-coding RNAs when cells were exposed to the drug.

Firstly, the global expression profile of lncRNAs and mRNAs for four different nigericin-treated time points was determined by a custom sequencing platform. By venn analysis, our data confirmed that 76 common dys-regulated lncRNAs including 49 up-regulated and 27 down-regulated ones might participate in the process of nigericin damage. These lncRNAs were widely distributed on all chromosomes except for sex chromosome X. Meanwhile, the common differentially expressed mRNAs among the 3 compared groups were also found, in which 172 mRNAs were common up-regulated and 85 ones were down-regulated. Subsequently, we chose 5 random lncRNAs and 5 cancer-related genes for PCR detection between the 0 and 32 h group. The data were consistent well with our sequencing data, which demonstrated the high reliability and validity of the sequencing expression results. Of these common differentially expressed mRNAs, GADD45A was found to be variously expressed in cell lines derived from PC, and adenoviral-mediated expression of GADD45A (Ad-G45a) in these cells resulted in apoptosis via caspase activation and cell-cycle arrest in the G2/M phase (32). HMG-box transcription factor 1 (HBP1) had been described as a negative regulator of the Wnt/β-catenin signaling in many cancers, including breast cancer (33), osteosarcoma (34), glioma (35), and colorectal carcinoma (36). A recent study by Chan also indicated that HBP1 acted as a direct downstream target of FOXO1, and potently suppressed the phenotypes of oral cancer (37). Besides, other 3 validated genes (SESN2, SIK1, and KIF20A) were also proved to influence the proliferation, migration and invasion of PC cells (38–41). These results might provide clues to the potential mechanisms of nigericin in PC.

Next, we conducted GO and KEGG pathway analyses to uncover the roles of these common differentially expressed mRNAs after nigericin treatment. The top 10 GO biological processes such as uridine catabolic process, nucleotide catabolic process and regulation of interleukin-6 biosynthetic process were found in the nigericin damage. Meanwhile, the differentially expressed mRNAs were significantly enriched in top 20 KEGG signaling pathways, including Aldosterone-regulated sodium reabsorption, Circadian rhythm, Mismatch repair, Drug metabolism-other enzymes, TNF signaling pathway, Transcriptional misregulation in cancers, TGF-beta signaling pathway, PI3K-Akt signaling pathway and so on. Moreover, the network between all KEGG pathways and their corresponding genes was also analyzed. These nigericin-related pathways have been also reported in PC. For example, the PI3K/Akt signaling pathway is related with PC metastasis. Tanno et al. showed that increased insulin-like growth factor I receptor expression induced by active Akt markedly enhanced the invasiveness of human PC cells (42). A recent review from Murthy et al. also described the role of PI3K signaling in PC development and progression (43). In 2014, Zhu et al. provided valuable baseline information regarding the TGF-β pathway in PC, which could be utilized in targeted therapy clinical trials (44). These involved non-coding RNAs (lncRNAs and mRNAs) and GO/KEGG analyses might partly explain the phenomena that nigericin had the anti-cancer properties.

To better understand the mechanisms of nigericin in PC cells, we built the co-expression network between lncRNAs and mRNAs. The network implied a complex relationship that one gene could correlate with multiple lncRNAs and one lncRNA might also regulate numerous mRNAs in different ways. For instance, up-regulated lnc-AGRN-2_9 was positively correlated with HBP1, GADD45A, SIK1, and SESN2, and negatively associated with TOP2A, CKAP2, while these mRNAs were implicated in tumorigenesis (32, 37–41, 45). The co-expression network might imply the potential regulatory mechanisms between lncRNAs and mRNAs in the nigericin anti-cancer process.

It has been known that lncRNAs can cis-regulate the co-expressed and nearby coding genes (24). In this study, we constructed a cis-regulated network with the criterion that coding genes located at 100 k bp upstream and downstream of lncRNAs on the chromosome. Our results showed that 6 of 76 common differentially expressed lncRNAs possessed cis-regulated genes, and each of the 6 lncRNAs only had one neighboring protein-coding gene. For example, we found that lnc-AGRN-2_3 and lnc-AGRN-2_9 shared the same cis-regulated gene MPV17L, which indicated that these two lncRNAs might play a similar role. Lnc-C9orf82-2_1 cis-regulated ADAMTS1, and Masui et al. also suggested that ADAMTS1 was a potential biomarker to detect early-stage PCs (46). UNC50 has long been recognized as a Golgi apparatus protein in yeast, and is involved in nicotinic receptor trafficking in Caenorhabditis elegans. In 2015, Fang et al. found that UNC50 was correlated with G1/S transition and proliferation in hepatocellular carcinoma via the influencing epidermal growth factor receptor trafficking (47). Interestingly, our data showed that UNC50 was involved with the nigericin damage, which could be cis-regulated by lnc-SLC25A3-3_1. These results revealed the prevalence of lncRNA-mediated cis-regulations on nearby genes during the nigericin damage.

On the other hand, previous reports have indicated that lncRNAs are capable of binding to a specific site or sequence, including TFs, to achieve trans-regulation functions. We constructed a TF-lncRNA binary network combined by these common differentially expressed lncRNAs with TFs. The network showed that 44 up-regulated lncRNAs were found to correspond to 31 TFs, and 27 down-regulated lncRNAs corresponded to 12 TFs. Furthermore, we introduced target genes to build TF-lncRNA-gene ternary network. 10 up-regulated lncRNAs correspond to 3 TFs and 283 target genes, while 6 down-regulated lncRNAs were found to associate with 3 TFs and 125 target genes. Interestingly, up to 14 dys-regulated lncRNAs were regulated by 5 TFs, such as MYC, TAF1, E2F4, STAT1, and STAT2. Recent evidence strongly suggests that these 5 TFs potentially regulate the expression of target genes in PC or other cancers. For instance, Valenti et al. found that Mutp53 and E2F4 proteins formed a transcriptional repressive complex that assembled onto the regulatory regions of BRCA1 and RAD17 genes inhibiting their expressions in head and neck squamous cell carcinoma (48). Guerrero-Zotano et al. identified 18 of the 20 E2F4 target genes, and suggested a potential benefit of adjuvant CDK4/6 inhibitors in patients with ER^+^ breast cancer who failed to respond to preoperative estrogen deprivation (49). STAT1, which is a member of the family of signal transducers and transcription activators, corresponded to lymph node metastasis, advanced stage, tumor dedifferentiation and poor prognosis in patients with PC (50). A study from Seshacharyulu et al. also confirmed STAT1 as a key regulator through down-regualtion of MUC4 in PC (51). Thus, our cis- and trans-regulation predictions might provide a deep insight into the involved lncRNAs in nigericin treatment.

Finally, a PPI network with common differentially expressed genes, in which 12 hub proteins were identified, including TOP2A, MYC, ANAPC1, FBXW7, KIF20A, MTOR, CREB1, EXO1, MELK, NEDD4L, RACGAP1, and HERC2. The most significant hub proteins were TOP2A and MYC. TOP2A could induce tumor development and progression in many cancer types, including PC (52), prostate cancer (53) and breast cancer (54). In 2016, a phase II study by Tarpgaard et al. found that metastatic colorectal cancer (mCRC) patients, who were refractory to treatment with oxaliplatin-based chemotherapy, had TOP2A gene amplification in their tumor cells (55). Similarly, human estrogen receptor-positive breast cancer cells typically displayed elevated levels of Myc protein due to overexpression of MYC mRNA (56). Other studies had also identified the abnormal expression of MYC-binding protein (MYCBP) during tumorigenesis in multiple types of cancer, such as gastric cancer (57), colon cancer (58), and PC (59). Therefore, this core PPI network exhibited the associations between these interested genes, which might provide useful clues for the mechanism analysis of nigericin in PC.

## Conclusion

In summary, our experiments further investigated the anti-cancer properties of nigericin in PC. In light of the high-throughput RNA sequencing analysis, we comprehensively characterized the potential contributions of lncRNAs and mRNAs after nigericin exposure. Additionally, the bioinformatics analyses, including GO and KEGG analysis, coding and non-coding co-expression network, cis- and trans-regulation predictions and PPI network, were applied to annotate the potential regulatory mechanisms among these coding and non-coding RNAs during the nigericin anti-cancer process. Our data provided new insight into the molecular mechanism of nigericin toward cancer cells, and suggested a possible clinical application in PC.

## Data Availability Statement

The datasets presented in this study can be found in online repositories. The names of the repository/repositories and accession number(s) can be found below: the NCBI Sequence Read Archive (https://www.ncbi.nlm.nih.gov/sra, PRJNA543685).

## Author Contributions

ZX conceived the project and wrote the manuscript. QZ and YX reviewed the manuscript. All authors participated in experiment and data analysis.

## Conflict of Interest

The authors declare that the research was conducted in the absence of any commercial or financial relationships that could be construed as a potential conflict of interest.
